# Supplementary figures and images for: rtpcr: a package for statistical analysis and graphical presentation of qPCR data in R
Source: PeerJ. 2025 Oct 13;13:e20185. doi: 10.7717/peerj.20185 (PMC12530193; doi:10.7717/peerj.20185)

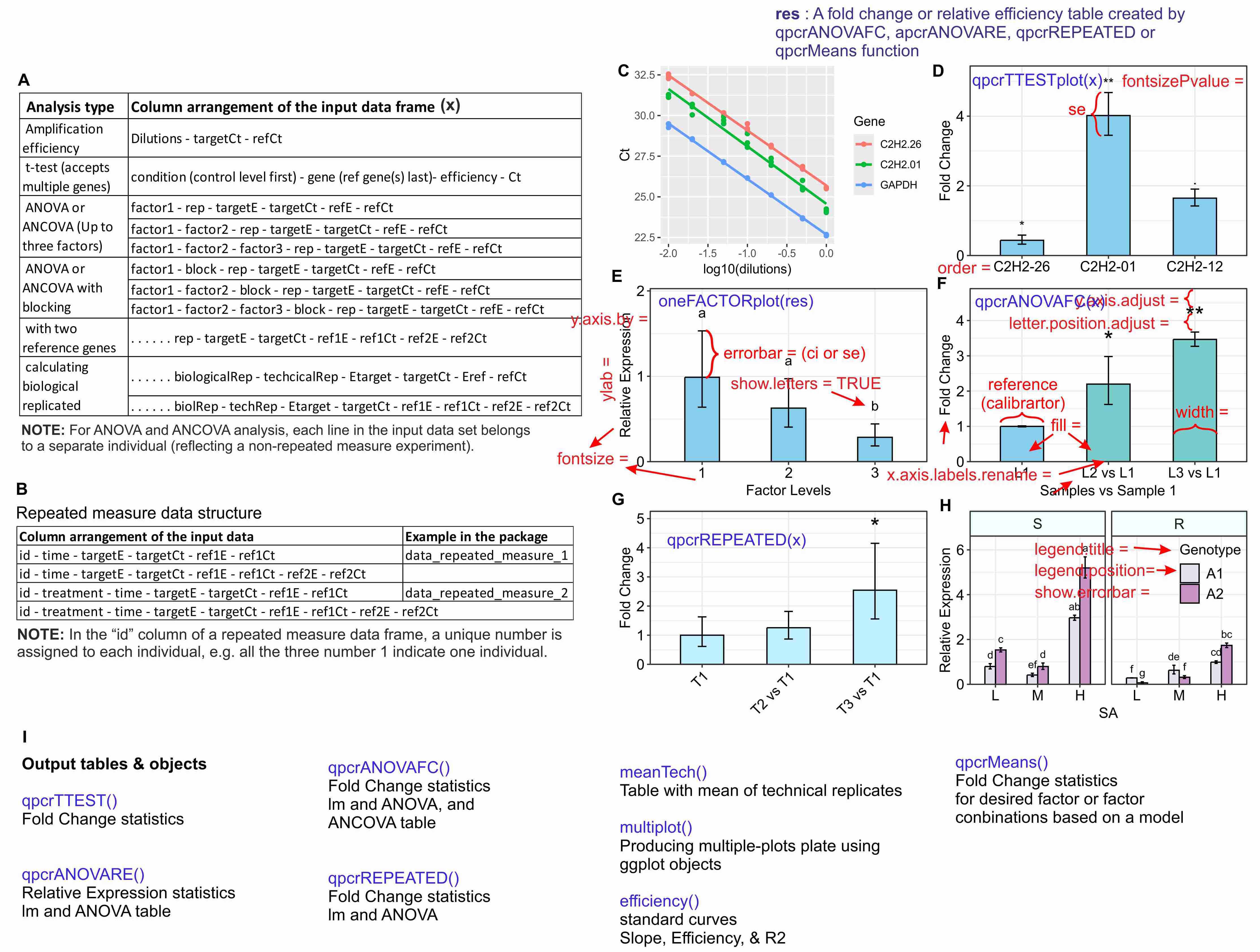

Supplement: Supplemental Information 1 — The used functions are presented in blue. (A) Standard curve and the amplification efficiency analysis of genes. (B) Average fold changes of three target genes relative to the control condition computed by unpaired t-tests via the ‘qpcrTTESTplot’ function. (C) Relative expression of a gene under three levels of a factor generated using the ‘oneFACTORplot’ function; (D) Plot of average Fold changes produced by the ‘qpcrANOVAFC’ function from the same data as ‘C’ where the level 1 has been selected as calibrator. The calibrator level can be selected by user. (E and F) Fold change and Relative expression of a target gene under two or three factors produced by ‘qpcrREPEATED and ‘threeFACTORplot’ functions, respectively. (G) List of output tables and objects from different functions. Error bars can be standard deviation or confidence interval. (H) Main output objects of some of the main functions of the rtpcr package. FC, foldchange (D D C_T method); RE, relative expression (D C_T method). [file peerj-13-20185-s001.jpg]
